# Evaluation of a phase-based motion tracking method for the calculation of myocardial stress and strain from tagged MRI

**DOI:** 10.1186/1532-429X-16-S1-P365

**Published:** 2014-01-16

**Authors:** Lennart Tautz, Anja Hennemuth, Teodora Chitiboi, Ulrich Kramer

**Affiliations:** 1 Fraunhofer MEVIS, Bremen, Germany; 2 University Hospital Tübingen, Tübingen, Germany

## Background

Tagged MRI is an established technique for the tracking of local deformations of the myocardium. The quantitative assessment of myocardial stress and strain is however a non-trivial task and only few software tools are available for clinical use. The purpose of this work was the evaluation of a phase-based motion tracking method for fully automatic calculation of deformation parameters from tagged MRI sequences [1].

## Methods

We calculated motion fields which form the basis for the computation of myocardial stress and strain for 40 single-slice tagged MR image sequences of healthy subjects. Image data was acquired at two different sites with four different MR scanners (Siemens Espree 1.5T, Avanto 1.5T, Trio 3T and Biograph 3T). Tagging grid spacing ranged from 6 to 8 mm, and in-plane resolutions from 1.2 to 1.6 mm. Images were oriented in short-axis (39) and 2-chamber view (1). The phase-based motion tracking approach estimates local deformation based on the application of quadrature filters [2]. The resulting motion field can then be used for motion tracking as well as for the calculation of stress and strain. For the assessment of the motion field quality, tracked landmarks were compared with expert observations. Two observers placed corresponding landmarks on tagging line crossings in 5 (2CH) or 6 (SAX) AHA segments of the myocardium in all image sequences. Inter-observer difference as well as deviation from the automatically tracked landmarks was calculated.

## Results

Calculation time of the automatic motion tracking algorithm was 39.13 ± 36.98 s on average per data set. The average deviation of the tracked landmark positions from the observer annotations was 0.43 ± 0.35 mm, and the average inter-observer difference was 1.17 ± 1.43 mm. The highest errors occurred for timeframes with rapidly moving myocardium and temporarily hidden tag lines due to throughplane motion.

## Conclusions

Altogether, automatic tracking error was in the order of magnitude of the voxel resolution, and is lower than the inter-observer difference. The evaluation shows that the method is capable of quantifying motion from tagged MRI data as well as manual observers. We thus assume that the method is suited for the calculation of clinically relevant parameters, such as circumferential and radial strain. The presented method is integrated into an OsiriX plugin that has been made available for clinical research.

## Funding

This work was partially funded by the European Regional Development Fund.

**Figure 1:**
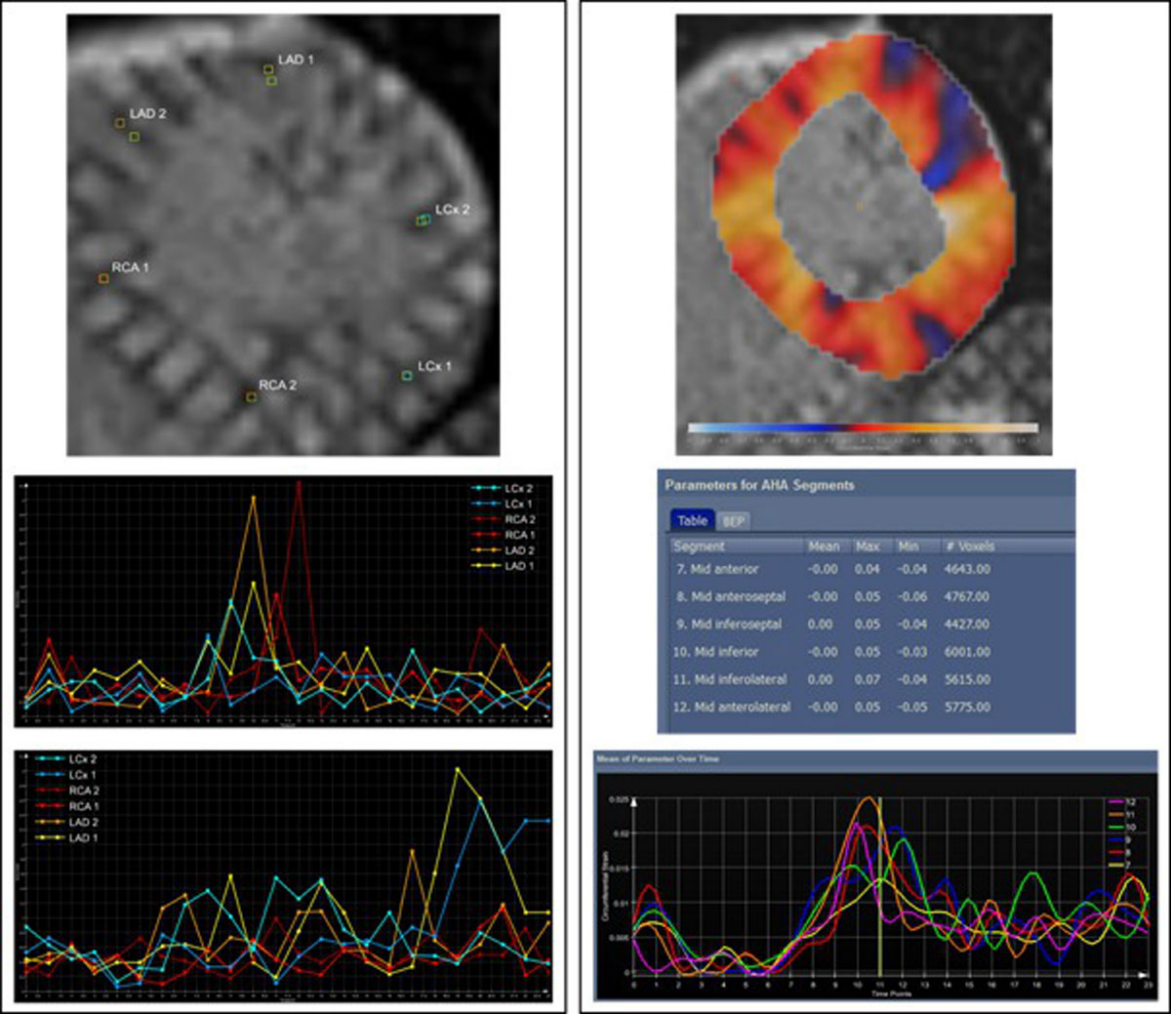
**Left (top to bottom): Tracked landmarks vs. observer landmarks, motion tracking deviation, inter-observer difference, Right (top to bottom): Circumferential strain: color overlay, parameters per AHA segment, parameter curves**.
